# Research progress on the role of the NEIL family in cancer

**DOI:** 10.3389/fcell.2025.1612329

**Published:** 2025-07-21

**Authors:** Yinghan Chen, Muyun Ma, Aixue Zou, Xinjia Wang, Weiwei Dong

**Affiliations:** ^1^Department of Obstetrics and Gynecology, Shengjing Hospital of China Medical University, Shenyang, China; ^2^Department of Rheumatology and Immunology, Beijing Hospital, Chinese Academy of Medical Sciences, Beijing, China; ^3^Department of Pediatrics, Clinical Medicine, China Medical University, Shenyang, China; ^4^Department of Neurosurgery, Shengjing Hospital of China Medical University, Shenyang, China

**Keywords:** NEIL, cancer, DNA, glycosylases, malignancy, treatment

## Abstract

Cancer is the primary cause of death worldwide. Cancer occurrence and progression are closely associated with DNA damage repair. DNA glycosylase were the first enzymes to initiate base excision repair, and the Nei-like DNA glycosylase (NEIL) family has attracted increasing attention as an important component of DNA glycosylases. Here, we introduce the role of the NEIL family in the malignant biological behaviors of cancer, including cell proliferation, chemoradiotherapy resistance, invasion and migration, apoptosis, and stemness. Mechanisms affecting the expression of the NEIL protein family range from the transcriptional level and mRNA editing to the level of post-translational modification. Additionally, we emphasize the different functions of the NEIL family in various malignancies and present useful information that supports the hypothesis that the NEIL family could be a potential target in the treatment and diagnosis of various cancers.

## 1 Introduction

Cellular DNA is persistently exposed to exogenous and endogenous damage (De Bont and van Larebeke, 2004; Hopkins et al., 2022; Bednarski and Sleckman, 2019). Exogenous sources of injury include ultraviolet light, environmental toxicants, and ionizing radiation (Hoeijmakers, 2001). Whereas, endogenous damage is predominantly caused by reactive oxygen species (ROS), including hydrogen peroxide (H_2_O_2_), the hydroxyl radical, ·OH, and the superoxide radical, O_2_^−^, and these compounds are important genotoxic substances (Hazra et al., 2002a). The accumulation of ROS can lead to oxidative damage and strand breaks in DNA, which are major threats to genome integrity (Sarker et al., 2021; Caliri et al., 2021; Seçme et al., 2023). Approximately 10^4^–10^5^ spontaneous DNA damage occurs in each cell every day (Yousefzadeh et al., 2021). Unrepaired damage may block DNA replication and transcription, and cause base mismatches, leading to aging and cancer (Chen et al., 2022; Yuan et al., 2023). Therefore, timely repair of DNA damage is vital for maintaining genome integrity.

Base excision repair (BER) is the primary pathway for repairing oxidative DNA damage (Hua and Sweasy, 2023). Specific DNA glycosylases that recognize and eliminate base damages initiate the BER pathway (Wiederhold et al., 2004). There are 11 DNA glycosylases in humans that are classified into three groups: Nei-like DNA glycosylases (NEILs, including NEIL1, NEIL2, and NEIL3), monofunctional enzymes and bifunctional glycosylases (Grundy and Parsons, 2020; Morland et al., 2002; Eckenroth et al., 2021; Hanna et al., 2021). After recognition, monofunctional enzymes catalyze the hydrolysis of the n-glycosidic bond, thereby removing damaged nitrogen bases and leaving an apurinic/apyrimidinic site. Then, apurinic/apyrimidinic nucleic acid endonuclease is recruited in order to hydrolyze the DNA backbone, forming a single strand break (SSB) with 5′ -deoxyribosephosphate and 3′-hydroxyl ends (Panigrahi et al., 2015; Gohil et al., 2023). In addition to cutting damage bases, bifunctional enzymes also exhibit innate apurinic/apyrimidinic cleavage enzyme activity, using β-elimination to generate 3′-αβ unsaturated aldehyde (Krokeide et al., 2013). In contrast, the NEIL family exhibits β,δ-elimination activity, creating a nucleotide gap with 3′-P and 5′-P termini (Dou et al., 2003). However, NEIL3 predominantly acts as a single functional enzyme using β-elimination in the presence of apurinic/apyrimidinic nucleic acid endonuclease, as a result of its weak apurinic/apyrimidinic lyase but vigorous glycosylase activity (Krokeide et al., 2013). DNA polymerase II repairs DNA, and ultimately, DNA ligase IIIa/X-ray repair cross complementing 1 completes the BER process (Tomkinson and Sallmyr, 2013).

**FIGURE 1:**
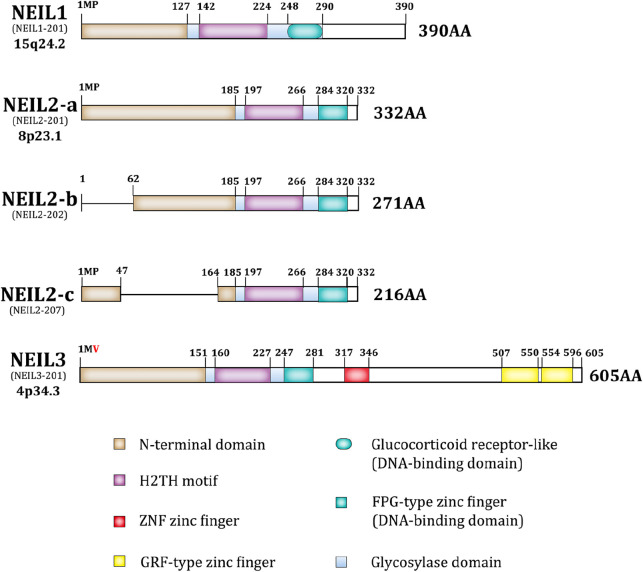
The structure of NEIL family.

As previously reported, NEIL1/2/3, nth like DNA glycosylase 1 (NTH1), and 8-oxoguanine DNA glycosylase (OGG1) are bifunctional; however, the NEIL family differs from the others (Figure 1). It has a complete Fpg/Nei core protein structural domain at the N-terminus, including an N-terminal domain, a helix-2-turn-helix (H2TH) motif, and a zinc finger or zinc-like finger DNA-binding motif (Das et al., 2004; Hazra and Mitra, 2006; Kladova et al., 2019). The NEIL family proteins exhibit both shared and distinct features in their structural conformations and substrate specificities. NEIL1 and NEIL2 share a conserved N-terminal region with the Fpg/Nei family, characterized by the presence of the H2tH motif that forms an αG helix. NTH1 and OGG1 utilize an innate Lys residue as the active site nucleophile, while NEIL1, NEIL2 uses an N-terminal Pro (Hazra et al., 2002a; Hazra et al., 2002b). NEIL1 is primarily involved in DNA replication and the excision of various oxidative lesions, including thymine glycol, 2,6-diamino-4-hydroxy-5-formamidopyrimidine, 5-hydroxycytosine, 5-hydroxyuracil, and 4,6-diamino-5-formamidopyrimidine. In contrast, NEIL2 features a unique C-terminal zinc finger motif and demonstrates a preference for lesions in single-stranded DNA and bubble structures. Interestingly, in NEIL3, Val replaces the vital catalytic role of Pro in the N-terminal structural domain. NEIL3 is nearly twice as large as other Fpg/Nei family members (Liu et al., 2013) and includes a duplicated Glycine arginine phenylalanine GRF-zinc finger (GRF-ZF) motif and a RANbp-like zinc finger (ZNF) motif at the C-terminus, which makes NEIL3 distinct from NEIL1 and NEIL2 (Rodriguez et al., 2020; Ha et al., 2020; Huskova et al., 2022). Although NEIL3 is less studied than NEIL1 and NEIL2, this enzyme displays broad substrate specificity, targeting both single-stranded and double-stranded DNA.

The disordered C-terminal domain of NEIL1 may be associated with multiple NEIL1 interactions. Although it has no effect on apurinic/apyrimidinic lyase activity or lesion excision, it is required for efficient enzymatic activity (Hegde et al., 2013b; Hegde et al., 2012; Hegde et al., 2015). Typically, the catalytic core of BER enzymes is an inflexible mold for detecting distorted DNA, whereas the catalytic structure of NEIL2 is more specialized in that the two lobes of its catalytic core are not in a catalytically-active orientation, making NEIL2 inactive by default until the catalytic domains are clustered together in the correct orientation. This structure may confer more function to NEIL2 (Tsutakawa and Sarker, 2021; Zhdanova et al., 2022; Eckenroth et al., 2023). In general, the unique structures of the NEIL family lead to diverse substrates and excise ring-opened or damaged oxidized pyrimidines and purines (Chakraborty et al., 2015; Makasheva et al., 2020).

The unifying feature of the NEIL family is the Fpg/Nei-like core protein structural domain (Glycosoylase domain), including an N-terminal domain, a helix-2-turn-helix (H2TH) motif, and a DNA-binding domain. NEIL3 is unique in that it is twice as large as the other NEIL proteins, having a RANbp-like zinc finger motif and a duplicated Glycine arginine phenylalanine GRF-zinc finger (GRF-ZF) motif at the C-terminus. Besides, in NEIL3 valine replaces the central catalytic role of proline in the second amino acid. The ensembl transcript ID and the location of the gene is under each isoform name, and the protein length is at the end of each isoform, as shown in Figure 1.

NEIL1 and NEIL3 are mainly active in the S phase and are involved in the repair of damaged bases prior to replication, whereas NEIL2 expression is cell cycle-independent (Hazra et al., 2002a; Albelazi et al., 2019; Neurauter et al., 2012; Hegde et al., 2013a). As multifunctional DNA repair enzyme, NEILs are crucial in diverse cellular processes. NEIL2 participates in active DNA demethylation (Schomacher et al., 2016), inflammatory responses (Tapryal et al., 2021), transcription-coupled repair (Chakraborty et al., 2021) and maintenance of the mitochondrial genome (Sarker et al., 2021; Mandal et al., 2012). NEIL3 prefers ssDNA-derived base damage, maintenance of replication fork stability, repair of DNA damage in G-quadruplex structures, repair of DNA interstrand crosslinks (ICL) and repair of telomere damage (Chen et al., 2022; Li et al., 2022; Li et al., 2020; Wu et al., 2019). The NEIL family plays a critical role in DNA repair, and the overexpression and downregulation of NEILs may be associated with cancer, which we will elaborate in the following sections.

## 2 Common targets and cellular functions of NEILs in various cancer

Growing evidence suggests that the NEIL family is involved in several cellular events such as proliferation, chemoradiotherapy resistance, migration, invasion, cell death, and stemness.

### 2.1 Cell proliferation

According to previous studies, NEIL proteins act as tumor suppressors or oncogenes during cell proliferation under different circumstances. High NEIL1 expression, for example, may be associated with a poor prognosis in patients with gastric cancer. The DNA repair inhibitor, berzosertib, may inhibit the expression of NEIL1, thus limiting the proliferation of gastric cancer cells (Ni et al., 2019). However, this is not the case in breast cancer or multiple myeloma. One study has suggested that decreased NEIL1 expression predicts poor survival outcomes in patients with invasive breast cancer (Shinmura et al., 2016). In melphalan-resistant multiple myeloma, NEIL1 expression is downregulated, leading to a greater propensity of cells to repair toxic ICL, thereby reducing G2/M phase arrest (Sousa et al., 2013). NEIL3 expression is limited to cells with a high capacity for proliferation (Fleming et al., 2015), and is inhibited in non-dividing cells (Neurauter et al., 2012). The most interesting aspect of NEIL3 is that it can repair telomeres (Zhou et al., 2013; Zhou et al., 2015; Karlsen et al., 2022; Fouquerel et al., 2016). NEIL3 expression is upregulated in non-small-cell lung cancer (NSCLC) (Huang and Hua, 2022). *In vitro*, NEIL3 protects the genome by repairing oxidative damage to telomeres in the S/G2 phase, which plays a significant role in the proliferation of NSCLC cells (Zhou et al., 2017). In hepatocellular carcinoma (HCC), NEIL3 repairs oxidative damage to telomeres and prevents telomere shortening during mitosis (Zhao et al., 2021b). However, NEIL3 does not influence prostate cancer (PCa) cell proliferation *in vitro* (Wang et al., 2021b). Studies have suggested that NEIL3 promotes the progression of lung and liver cancers by regulating PI3K/Akt/mTOR signal transduction (Huang and Hua, 2022; Wang et al., 2021). These findings suggest that NEILs play a dual role in cancer that is specific to the cellular environment.

### 2.2 Chemotherapy resistance

Acquired chemotherapy resistance is a significant cause of death in patients with advanced malignant tumors; therefore, there is an urgent need to identify the mechanisms of chemotherapy resistance (Davodabadi et al., 2023). NEILs play a pivotal role in the resistance of PCa, HCC, and other tumors to chemotherapy.

During chemotherapy, NEIL3 confers resistance to cisplatin, which is one of the most commonly used chemotherapeutic drugs in the treatment of a wide range of solid tumors (Rottenberg et al., 2021). Cisplatin induces DNA ICL, which blocks DNA replication and transcription, and eventually triggers apoptosis, which is the main mechanism against tumors. NEIL3 is one of the main enzymes involved in ICL repair (Semlow and Walter, 2021; Yang et al., 2017; Chung et al., 2023; Imani Nejad et al., 2020).

Neuroendocrine prostate cancer, castration-resistant prostate cancer (CRPC), and metastatic PCa are all considered advanced PCa (Wang et al., 2018). NEIL3 knockdown markedly decreased the sensitivity of PCa cells to cisplatin in two ways (Wang et al., 2021c). On the one hand, the knockdown of NEIL3 reduces cell cycle arrest in the S phase, and on the other hand, it regulates ataxia-telangiectasia mutated (ATM) and ataxia-telangiectasia and Rad3 related (ATR) pathway activities. NEIL3 itself does not affect the levels of phosphorylated ATM (p-ATM) or phosphorylated ATR (p-ATR); however, upon exposure to docetaxel or cisplatin, the phosphorylation of ATM and ATR was significantly promoted by the loss of NEIL3, thereby triggering downstream pathways associated with DNA repair.

Resistance to chemotherapy may be promoted by the activation of epithelial-mesenchymal transition (EMT), which enhances survival mechanisms and cell cycle progression (Marie-Egyptienne et al., 2013). In HCC, NEIL3 activates a key transcription factor, twist family bHLH transcription factor 1 (TWIST1), leading to an increase in the expression of the drug efflux gene and drug resistance in EMT (Lai et al., 2022).

In HeLa cells, NEIL3 interacts with WRN helicase interacting protein 1 (WRNIP1) through its C-terminal domain to target WRNIP1 to the proteasome, promotes WRNIP1 degradation and ICL repair, and induces cisplatin resistance (Aliyaskarova et al., 2023). WRNIP1 is a member of the AAA ATPase family and involved in the early stages of ICL repair (Socha et al., 2020). Its timely degradation at a later stage of repair can cause the ICL repair to proceed to the next step.

NEIL1 can also induce cisplatin resistance via a mechanism that is different from that of NEIL3. It has been suggested that BER maintains cisplatin cytotoxicity by decreasing ICL repair through competition with the cisplatin ICL DNA repair pathway (Kothandapani et al., 2011). In multiple myeloma cells, NEIL1 depletion contributes to melphalan resistance by downregulating the BER pathway, which facilitates the repair of the more toxic ICL (Sousa et al., 2013).

A defect in single-stranded DNA damage repair is considered a new factor that causes endocrine resistance in ER+ breast cancer. It has been reported that the loss of NEIL2 leads to endocrine resistance via disruption of the G1-S phase transition, but more specific mechanisms remain to be elucidated (Anurag et al., 2018). NEIL2 is involved in the regulation of cellular ROS concentrations in breast cancer stem cells. Its high expression repairs ROS-induced DNA damage, maintains ROS at low levels, and leads to resistance to doxorubicin and other chemotherapeutic drugs that produce ROS as the primary killing mechanism (Banerjee et al., 2020).

### 2.3 Other functions

Migratory and invasive cells have increased capacity during tumor development, which is closely related to metastasis at advanced stages of cancer. Various mechanisms are involved in the acquisition of these malignant features by tumor cells. EMT in hepatoma may be promoted by NEIL3 through activation of the BRAF/MEK/ERK/TWIST signaling pathway (Lai et al., 2022). NEIL3 directly induces BRAF transcription, which activates the downstream mitogen-activated protein kinase (MAPK) cascade of BRAF, phosphorylates ERK, and activates the downstream molecule, TWIST1, thereby promoting EMT. EMT is an important step in tumor metastasis (Zhu et al., 2016; Manfioletti and Fedele, 2022). Interestingly, NEIL3 promotes EMT independent of DNA repair, which was not observed for NEIL1 and NEIL2 in this study. NEIL3 can also partially promote the proliferation, invasion, and migration of NSCLC cells by regulating the classical PI3K/AKT/mTOR signaling pathway (Huang and Hua, 2022).

NEIL target genes also participate in apoptosis regulation. NEIL1 inhibits apoptosis by increasing the expression of the anti-apoptotic gene, BCL2 apoptosis regulator (Bcl-2), and decreasing the expression of pro-apoptotic genes (BCL2 associated X, apoptosis regulator (Bax) and caspase-9) in colorectal cancer (CRC) cells (Xue et al., 2020). NEIL2 expression level in cells is related to ROS levels. In breast cancer cells, low concentrations of ROS induce the upregulation of NEIL2 and enhance BER; however, high concentrations of ROS cause a decrease in NEIL2, which results in the activation of p53 and further activates the intrinsic apoptotic pathway (Banerjee et al., 2020; Chakraborti et al., 2017). NEIL3 also inhibits apoptosis by repairing gene damage (Wang et al., 2021c).

Maintenance of stemness is another malignant biological behavior of tumors. Breast cancer stem cells employ several molecular strategies to evade chemotherapy-induced death signals, and redox regulation is a key factor. High levels of NEIL2 expression can maintain low levels of ROS and stemness in breast cancer stem cells (Banerjee et al., 2020).

## 3 Common regulatory mechanisms for NEIL proteins in cancer

**FIGURE 2:**
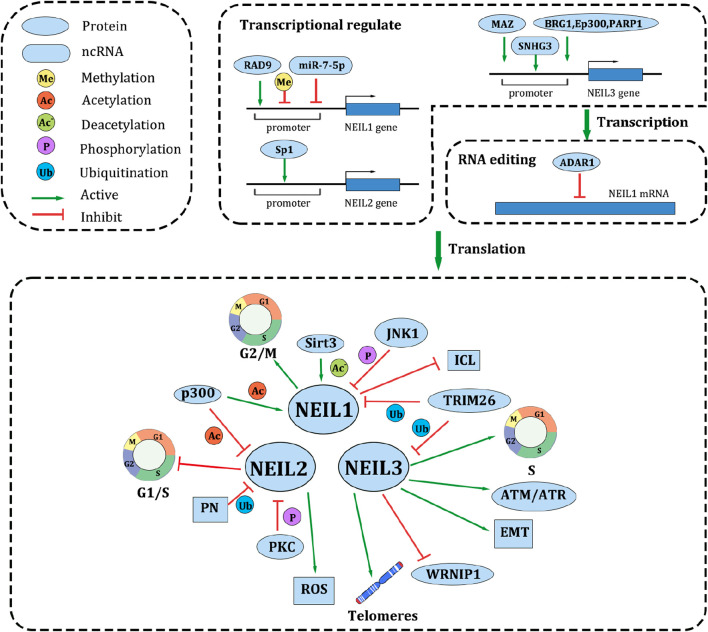
Common upstream regulatory mechanisms, targets and cellular functions of NEIL family. In the transcriptional level, promoter methylation, the combination of several proteins and ncRNAs may affect the NEIL family expression. Additionally, ADAR1 edits NEIL1 mRNA after transcription. After translation, a variety of protein modifications such as acetylation, deacetylation, phosphorylation, and ubiquitination affect the activity of NEIL protein. NEIL family also influence the malignant biological behaviors of cancer in several ways. Reducing cell cycle arrest and repairing telomeres are the main approaches to accelerate cell proliferation. In chemotherapy resistance, NEIL1 competes with ICL DNA repair pathway to maintains cisplatin cytotoxicity. NEIL2 promotes ROS repair, besides, loss of NEIL2 leads to disruption of the G1-S phase transition. NEIL3 promoting EMT pathway and ATM/ATR pathway, but inhibiting WRNIP. NEIL family also play a vital role in invasion and migration, apoptosis, and stemness.

The NEIL protein family is regulated by diverse mechanisms, including transcriptional, post-transcriptional, and post-translational regulation (Figure 2).

### 3.1 Transcriptional and post-transcriptional regulation

The regulation of NEIL at the transcriptional level is primarily mediated by its promoter. A subset of factors modulates the expression of the NEIL family by directly binding to their promoters. Aberrant RAD9 checkpoint clamp component A (RAD9) expression is associated with various cancers. In PCa DU145 cells, RAD9 binds to the NEIL1 p53 consensus sequence in the promoter region, promoting NEIL1 transcription. RAD9 is crucial in maintaining genome stability, acting primarily as part of the 9-1-1 complex. It also acts as a transcription factor independent of 9-1-1, regulating DNA repair, cell cycle checkpoints, apoptosis, and telomere stability (Broustas et al., 2019; Lieberman et al., 2017; Panigrahi et al., 2015; McDonald et al., 2023; Guan et al., 2007). Oxidative stress can regulate the expression of NEIL2 (Kinslow et al., 2010). Binding of Sp1 to the NEIL2 promoter is boosted by low ROS levels, thereby promoting NEIL2 transcription and activating DNA damage repair (Chakraborti et al., 2017). Pyridinol reduces NEIL2 expression while inhibiting its association with RNA Pol II, thereby impeding NEIL2-mediated transcriptional coupling repair, promoting DNA damage, and initiating ROS production (Banerjee et al., 2020). Single nucleotide polymorphisms (SNPs) in the NEIL2 promoter may also influence its expression (Kinslow et al., 2008). The transcription factor, MYC associated zinc finger protein (MAZ), functions as an upstream regulator of NEIL3 to directly promote its transcription and this induces cisplatin resistance in lung adenocarcinoma (Wang et al., 2023).

Non-coding RNAs can also affect NEIL transcription. Small nucleolar RNA host gene 3 (SNHG3), a long non-coding RNA, recruits the transcription factor, E2F1, to the promoter region of NEIL3 and activates its transcription in HCC (Zhang et al., 2023). In colon cancer, miR-7-5p targets the 3′UTR of NEIL to suppress NEIL1 transcription, thereby increasing anti-apoptotic genes, Bcl-2, down-regulating pro-apoptotic genes (Bax and caspase-9), and inhibiting apoptosis (Xue et al., 2020).

Notably, the modification of the promoter region is an important way to regulate NEIL expression. NEIL1 expression is downregulated by the hypermethylation of the promoter region of NEIL1 in head and neck squamous cell carcinoma (Chaisaingmongkol et al., 2012). BRG1 is a member of the SWI/SNF chromatin-remodeling complex that acts as an activator of functionally connected genes and promotes DNA repair and mitotic cell division (Orlando et al., 2019; Takao et al., 2017). The BRG1-EP300 complex drives NEIL3 transcription in breast cancer. EP300 acetylates nucleosomes in the promoter region, and BRG1 evicts acetylated nucleosomes from the chromatin, thereby promoting transcription. PARP1 co-localizes with BRG1 on the highly acetylated promoter of NEIL3, ensuring an open chromatin structure (Sobczak et al., 2020; Sobczak et al., 2019).

RNA editing can also alter NEIL protein expression. The RNA editing enzyme, adenosine deaminase (ADAR1), converts adenosine to inosine (A-to-I) (Avesson and Barry, 2014). It has been reported that there may be different A-to-I editing levels at the NEIL1 RNA (lysine 242 AAA codon) site (Lee et al., 2017; Minko et al., 2020; Lotsof et al., 2022). Unedited NEIL1 repairs DNA much more quickly than edited NEIL1 (Yeo et al., 2021; Yeo et al., 2010). ADAR1 amplified lung cancer cell lines show a higher frequency of AAA-AIA and AAA-to-AII edits than ADAR1 normal cells (Anadón et al., 2016). NEIL1 is a vital and extensively edited ADAR1 target in multiple myeloma. The recoded NEIL1 protein shows a loss of oxidative damage repair capacity and an increased cell growth rate (Teoh et al., 2018). These data provide new insights into the molecular pathogenesis of multiple myeloma at the mRNA level.

### 3.2 Post-translational modifications

Post-translational modifications are currently a popular research topic. Studies have shown that the BER is strictly modified after translation. The addition of an acetyl group to a lysine (K) residue is a critical post-translational modification that modulates the function of a protein by altering its conformation, activity, stability, or ability to interact with other proteins (Bhakat et al., 2020; Shvedunova and Akhtar, 2022). Research has shown that NEIL1 is acetylated at multiple K residues (K296, K297, and K298) and that NEIL1 acetylation enhances its glycosylation enzyme activity *in vitro*. Acetylation of NEIL1 only combined with chromatin (Bacolla et al., 2021). In addition, acetylation-defective NEIL1 protein enhances cell sensitivity to DNA-damaging agents that produce SSBs or oxidized bases. At the same time, acetylation can affect the interaction of NEIL1 with other proteins. Compared to wild-type NEIL1, the 3KR mutant, which is acetylation-defective, forms less stable complexes with diverse chromatin proteins, such as BER/SSB repair partners and histone chaperones. The BER activity of the repair complex bound to the 3KR mutant was remarkably lower than that of wild-type NEIL1. In conclusion, the primary role of acetylated lysine residues in NEIL1 is to stabilize the chromatin-binding repair complex formation, thus protecting cells from oxidative stress (Sengupta et al., 2018). The mitochondrial protein, sirtuin 3 (Sirt3), participates in various metabolic regulatory processes (Onyango et al., 2002). Sirt3 not only controls metabolism at the transcriptional level but also directly regulates the activity of metabolic enzymes. In CRC, deacetylation of NEIL1 and NEIL2 is regulated by Sirt3. Meanwhile, Sirt3 directly engages with NEIL1 (Kabzinski et al., 2022; Kabziński et al., 2019). *In vitro* results showed that NEIL2 was acetylated by p300 at K49 and K153. Acetylation of K153 does not affect NEIL2 activity *in vitro*, whereas acetylation of K49 inhibits NEIL2 base excision and apurinic/apyrimidinic decomposition (Bhakat et al., 2004).

Ubiquitination is a key regulatory mechanism in this process (Cockram et al., 2021). Studies have discovered two enzymes that catalyze NEIL1 polyubiquitination, tripartite motif 26 (TRIM26) and Mcl-1 ubiquitin ligase E3 (Mule). These enzymes can polyubiquitinate NEIL1 *in vitro*, and both catalyze the ubiquitination of NEIL1 at the same C-terminal lysine residue. TRIM26 and Mule are important for sustaining steady-state levels of NEIL1 and are required for DNA damage responses. NEIL1 may be induced by ionizing radiation and may contribute to ionizing radiation resistance after TRIM26 silencing (Edmonds et al., 2017). TRIM26 tightly regulates NEIL1 and NEIL3 levels and regulates the oxidative stress response induced by hydrogen peroxide (Konis et al., 2022). Another study showed that Pyridoxine (PN) reduced the protein level of NEIL2 by enhancing its ubiquitination and degradation (Banerjee et al., 2020).

Phosphorylation is another method of regulating NEIL protein activity. The phosphorylation of three serine residues in NEIL1 was revealed by mass spectrometry: S207, S306, and S61. Phosphorylation did not influence the enzyme activity or DNA binding at the three serine sites. However, the mutation of another phosphorylation site, Y263, to E, produced a completely inactive enzyme. C-Jun N-terminal kinase 1 (JNK1) is involved in NEIL1 phosphorylation. As a member of the MAPK family, JNK1 interacts with NEIL1 *in vitro* and phosphorylates residues, S207, S306, and S61 (Prakash et al., 2016). Another study showed that two kinases, protein kinase C (PKC) and cyclin-dependent kinase 5 (CDK5), phosphorylate NEIL2 in human SH-SY5Y neuroblastoma cells. The two kinases regulate NEIL2 function in different ways. CDK5 does not directly influence NEIL2 activity *in vitro*, whereas PKC phosphorylation of NEIL2 results in a significant decrease in NEIL2 repair activity. Interestingly, NEIL2 is rapidly dephosphorylated in response to oxidative stress in SH-SY5Y cells, suggesting that phosphorylation is a critical regulator of NEIL2 function, especially under oxidative stress (Myrup Holst et al., 2023).

## 4 NEIL proteins in cancer

The NEIL family is modified in diverse cancer types (Table 1). These alterations include dysregulation of gene expression and SNPs. Our review focuses on the cancer types in which NEIL family members were involved and for which sufficient data was available.

**TABLE 1:** Deregulation of NEILs in cancers.

Cancer type	NEIL member	Expression levels	Genetic alteration/Regulation	Association with cancer/Biological process	Proposed cancer role	Ref.
Lung	NEIL1	Low	Methylation	May be used as a potential therapeutic target	Tumor suppressor	Do et al. (2014)
Lung	NEIL1	Low	RNA edit	Patients with early stage lung cancer with RNA edit gene amplification have a poor prognosis	Tumor suppressor	Anadón et al. (2016)
Lung	NEIL1	Undescribed	SNP	May be used as independent biomarkers for predicting radiation pneumonitis	Undescribed	Zheng et al. (2021)
Lung	NEIL2	Low	miR-548a	Downregulation of miR-548a and upregulation of NEIL2 expression decreases NSCLC cell sensitivity to cisplatin	Tumor suppressor	He et al. (2020)
Lung	NEIL2	Low	Undescribed	The level of NEIL2 is extremely low in half of cancer tissues	Tumor suppressor	Sarker et al. (2014)
Lung	NEIL3	High	Undescribed	Repair DNA damage caused by telomerase and involve in the occurrence and development of lung cancer, promoting cisplatin resistance. May be related to worse clinical features and prognostic outcomes	Oncogene	Wang et al. (2023), Tran et al. (2020), Frías et al. (2008)
Prostate	NEIL1	High	Undescribed	May be involved in tumorigenesis and metastasis-associated phenotypes	Oncogene	Broustas et al. (2019), Panigrahi et al. (2015)
Prostate	NEIL3	High	Undescribed	May participate in the onset or migration of prostate cancer cells	Oncogene	Zhuang et al. (2022)
Prostate	NEIL3	Low	Undescribed	NEIL3 is downregulated in NEPC and CRPC cell lines, loss of NEIL3 contributes to chemoradiotherapy resistance	Tumor suppressor	Wang et al. (2021c), Wang et al. (2021b)
Breast	NEIL2	Low	Undescribed	Loss of NEIL2 leads to endocrine therapy resistance	Tumor suppressor	Anurag et al. (2018)
Breast	NEIL2	High	Undescribed	NEIL2 expression is elevated in bCSCs and may be associated with cancer risk in carriers of BRCA2 mutations	Oncogene	Banerjee et al. (2020), Osorio et al. (2014)
Breast	NEIL3	High	Undescribed	Play a role in the development and progression	Oncogene	Deng et al. (2022)
Liver	NEIL1	Undescribed	Undescribed	NEIL1 expression is impaired in HCV-infected cells and may protect cells from the mutagenic and cytotoxic effects of NM-Fapy-dG	Tumor suppressor	McCullough and Lloyd (2019), Pal et al. (2010)
Liver	NEIL3	High	Undescribed	Repairs telomere oxidative damage to prevents HCC senescence and may promote the migration, invasion, and stemness	Oncogene	Zhao et al. (2021b), Wang et al. (2021)
Colorectum	NEIL1	High	Undescribed	Enhances cell proliferation and reduces apoptosis	Oncogene	Xue et al. (2020)
Stomach	NEIL1	Low	Undescribed	Decreased expression may be associated with the pathogenesis of few gastric cancers	Tumor suppressor	Shinmura et al. (2004)
Stomach	NEIL2	Low	Undescribed	NEIL2 expression is markedly reduced by *H. pylori* infection	Tumor suppressor	Sayed et al. (2020b)

NEPC, neuroendocrine prostate cancer; bCSCs, Breast cancer stem cells; HCC, hepatocellular carcinoma; NSCLC, non-small-cell lung cancer; BRCA2, BRCA2 DNA, repair associated; NM-Fapy-dG, nitrogen mustard-formamidopyrimidine; HCV, hepatitis C virus; CRPC, castration-resistant prostate cancer.

### 4.1 NSCLC

With the second highest incidence of malignancy worldwide, NSCLC accounts for approximately half of all cancer-related deaths worldwide. Although considerable progress has been made in the diagnosis and treatment of NSCLC, the 5-year survival rate of patients with lung cancer remains less than 20%. Understanding the molecular mechanisms and potential therapeutic targets of NSCLC is urgently needed (Siegel et al., 2023; Herbst et al., 2018; Duma et al., 2019).

NEILs are crucial in the origin, progression, diagnosis, and treatment of NSCLC. The deletion, silencing, and abnormal expression of NEIL1 may play important roles in lung cancer pathogenesis. In one study, the incidence of lung tumors in Nth1^−/−^Neil1^−/−^ mice was much higher than that in single knockout Nth1^−/−^ or Neil1^−/−^ mice (Chan et al., 2009). The NEIL1 promoter is frequently methylated in NSCLC, making it a potential therapeutic target (Do et al., 2014). The adeno-inosine-editing enzyme, ADAR1, is genetically amplified in NSCLC cell lines and primary tumors, and its overexpression enhances tumorigenic potential in cell culture and mouse models. Functionally, ADAR1 overexpression increases the editing frequency of lysine 242 AAA codons A-to-I of the target transcript of NEIL1. In the clinical setting, patients with early stage lung cancer with ADAR1 gene amplification have a poor prognosis (Anadón et al., 2016). Novel therapies targeting specific gene mutations in NSCLC are promising in terms of improving patient survival. Oxidative stress plays an important role in the progression of lung cancer. Smoking dramatically increases oxidative stress, and carotenoids are potent antioxidants. Lower doses of lycopene improve the levels of NEIL1, NEIL2, and NEIL3 in cigarette smoke-induced A549 human lung cancer epithelial cells. These findings can help elucidate the molecular mechanisms underlying the anti-lung cancer action (Cheng et al., 2020). Radiotherapy plays a vital role in the treatment of thoracic tumors; patients with lung cancer who receive radiotherapy may experience radiation-induced lung injury, which can cause radiation pneumonitis (Giuranno et al., 2019). A total of 174 lung cancer patients treated with radiotherapy were genotyped for the NEIL1 genetic variants, rs4462560 and rs7402844. NEIL1 mutations were related to the risk of radiation pneumonitis by regulation of NEIL1 expression and acted as independent biomarkers for predicting radiation pneumonitis in patients treated with thoracic radiotherapy (Zheng et al., 2021).

NEIL2 polymorphisms may affect the development and treatment sensitivity of lung cancer. It has been reported that in patients with advanced NSCLC, progression free survival is associated with rs8191670 in the Neil2 gene, which is a polymorphism (T/C). The potential molecular mechanism may be that miR-548a decreases the expression of NEIL2 through binding to its 3′UTR which contains rs8191670. Downregulation of miR-548a and upregulation of NEIL2 expression decreases NSCLC cell sensitivity to cisplatin (He et al., 2020). In addition, DNA from the R257L variant (rs8191664) shows a lower affinity for other repair proteins, especially Polβ, leading to reduced repair capacity and increased endogenous DNA damage, which can eventually lead to lung cancer (Dey et al., 2012). In lung cancer, NEIL2 acts as a tumor suppressor, and the level of NEIL2 is extremely low in half of cancer tissues. Smoking and exposure to secondhand smoke play vital roles in the development of lung cancer. Impaired NEIL2 expression in sidestream smoke-exposed nonsmokers may cause the accumulation of genomic DNA mutations, which could lead to sidestream smoke-induced lung cancer (Sarker et al., 2014).

Unlike NEIL1 and NEIL2, NEIL3 functions as an oncogenic factor in lung cancer. NEIL3 can repair DNA damage caused by telomerase in the S phase and reduce the destructive effects of ROS. NEIL3 is upregulated in NSCLC tissues, suggesting that NEIL3 is involved in the occurrence and development of lung cancer. Clinical correlation and prognostic analyses revealed that NEIL3 is related to worse clinical features and prognostic outcomes (Frías et al., 2008; Tran et al., 2020). Therefore, NEIL3 may be a potential therapeutic target and prognostic predictor. The transcription factor, MAZ, increases NEIL3 expression and inhibits DNA damage in lung adenocarcinoma cells, thereby promoting cisplatin resistance in the lung adenocarcinoma cells (Wang et al., 2023). In addition, NEIL3 partially activates the PI3K/AKT/mTOR signaling pathway. NEIL3 level positively correlate with chemosensitivity to cisplatin and paclitaxel (Huang and Hua, 2022). Several studies have established NEIL3-related survival and prognostic models, providing new diagnostic and treatment strategies for NSCLC (Zhao et al., 2022; Ali et al., 2022; Zhao et al., 2021a; Ömeroğlu Şimşek et al., 2020).

Taken together, NEIL1 and NEIL2 function as tumor suppressors and their abnormal expression can lead to the development of lung cancer and drug resistance. NEIL3 functions as an oncogenic factor and its high expression is associated with poor prognosis and chemoresistance.

### 4.2 Prostate cancer

PCa is a complex condition that affects a wide range of men globally. With early detection and treatment, patients with local disease and a low-to-moderate risk of recurrence generally have a good prognosis. Current research is aimed at improving the diagnosis and treatment of PCa and understanding of the basic biological characteristics of the disease at all stages (Rebello et al., 2021; Wasim et al., 2022; Lowrance et al., 2023).

RAD9 regulates BER by influencing NEIL1 levels, and RAD9A plays a vital role in prostate tumorigenesis and metastasis-associated phenotypes (Broustas et al., 2019; Panigrahi et al., 2015). Unlike NSCLC, NEIL3 plays multiple roles in PCa. It may participate in the onset or migration of PCa cells; however, its deletion can lead to resistance to chemoradiotherapy. NEIL3 is downregulated in neuroendocrine prostate cancer and CRPC cell lines, and NEIL3 is associated with a high Gleason score, but a good prognosis. NEIL3 modulates the cell cycle by negatively regulating ATR expression. Loss of NEIL3 contributes to chemoradiotherapy resistance in PCa, and may be a potential target for patients with chemoradiotherapy resistance (Wang et al., 2021c; Wang et al., 2021b). NEIL3 is a central gene involved in the inhibition of PCa progression by the combination of aspirin and lipitor (Wang et al., 2022b).

Fonofos, an organophosphate insecticide, the interaction between fonofos and NEIL3 rs1983132 significantly increases the risk of PCa in patients with a family history of PCa (Barry et al., 2011). Moreover, NEIL3 variants may be associated with PCa (Kim et al., 2016; Liu et al., 2020; Yadav et al., 2020). NEIL3 can also promote PCa metastasis. The regulation of NEIL3 by FOXM1 may be a potential pathway for promoting the migration of prostate cells while participating in anti-androgen resistance in PCa (Zhuang et al., 2022). According to bioinformatics analyses, NEIL3 is a potential biomarker for the prediction and prognosis of PCa (Teng et al., 2021).

In conclusion, NEIL proteins are related to the occurrence, metastasis, and sensitivity to chemoradiotherapy in PCa, and can be used as markers to predict efficacy and prognosis.

### 4.3 Breast cancer

Breast cancer is a complex disease involving both genetic and environmental factors. Diverse treatments for breast cancer have been developed; however, drug resistance remains a major problem. Breast cancer stem cells are major contributors to aggressiveness and drug resistance, posing a major challenge in cancer treatment. Thus, the detection and prognosis of breast cancer need to be improved (Barzaman et al., 2020; Tsang and Tse, 2020; Britt et al., 2020).

NEIL2 and NEIL3 play a role in the development and progression of breast cancer. NEIL2 rs1466785 and rs804271 are associated with cancer risk in carriers of BRCA2 mutations (Osorio et al., 2014; Benítez-Buelga et al., 2017). Furthermore, increased NEIL2 expression enhances the sensitivity of breast cancer cells to double-strand breaks and apolipoprotein B mRNA editing enzyme catalytic subunit 3 (APOBEC3) deaminase-mediated mutations by interfering with BER (Shen et al., 2020). The BRG1-EP300 complex drives NEIL3 transcription in breast cancer. EP300 acetylates nucleosomes in the promoter region, and BRG1 evicts acetylated nucleosomes from the chromatin, thereby promoting transcription. PARP1 co-localizes with BRG1 on the highly acetylated promoter of NEIL3, ensuring an open chromatin structure (Sobczak et al., 2020; Sobczak et al., 2019). NEIL3 may also be involved in the tumorigenesis induced by estrogen and progestin therapy in breast cancer (Deng et al., 2022).

Therapeutically, the curcumin analog, 3,5-bis (4-hydroxy-3-methoxy benzylidene) -N-methyl-4-piperidone (PAC), induces apoptosis by upregulating Bax expression and downregulating Bcl-2 expression in triple-negative breast cancer cell lines. PAC also upregulates the expression of NEIL2. This opens up a new perspective for triple-negative breast cancer treatment (Almalki et al., 2023). Regulation of cellular redox status may also be a potential way to treat drug-resistant breast cancer. Peg-functionalized zno nanoparticles can generate ROS and exert anti-cancer effects. In breast cancer, the binding of Sp1 to the NEIL2 promoter is boosted by low ROS, thereby promoting NEIL2 transcription and enhancing BER. However, high concentrations of ROS lead to a decrease in NEIL2, which results in the activation of p53 and further activates the intrinsic apoptotic pathway (Chakraborti et al., 2017). Another study showed that PN enhances the chemotherapy responsiveness of breast cancer stem cells through redox modulation. NEIL2 expression is elevated in breast cancer stem cells, leading to doxorubicin resistance. However, vitamin B6 and PN, inhibit NEIL2-mediated transcriptional coupling repair processes by reducing NEIL2 expression and inhibiting its association with RNA *Pol II*, thereby stimulating DNA damage and triggering ROS production (Banerjee et al., 2020).

Research on the mechanisms underlying resistance to endocrine therapy has also progressed. Loss of NEIL2 leads to endocrine therapy resistance via dysregulation of the G1-S transition, and miRNA regulation of NEIL2 may mediate the prognosis of hormone-treated breast cancer (Anurag et al., 2018). The SNPs of miRNA binding sites (miRSNPs) in the 3′-untranslated region of NEIL2 may affect the binding affinity of miRNA. For example, patients with genotype of NEIL2 rs6997097 who received only hormone therapy had significantly shorter disease-free survival and overall survival (Cumova et al., 2021). In addition, NEILs may serve as risk indicators of breast cancer (Matta et al., 2013).

In conclusion, NEIL2 may provide new perspectives for breast cancer treatment, and NEIL3 may have a role in the onset and progression of breast cancer.

### 4.4 Hepatocellular carcinoma

HCC is one of the most common cancers and a major global healthcare challenge (Vogel et al., 2022; Yang et al., 2019; Zhou and Song, 2021). The NEIL family is associated with liver cancer caused by hepatitis virus and aflatoxins (Li et al., 2017). Dietary exposure to aflatoxi (AFB1) and subsequent DNA damage are important promoters. Currently, there are several treatment options for HCC, and there is an urgent need to identify predictive biomarkers to inform treatment selection.

NEIL1 expression is impaired, whereas NEIL2 expression is unaffected in HCV-infected cells (Pal et al., 2010). Alkylation damage of DNA bases can be caused by diverse drugs, such as AFB1 and chemotherapeutic nitrogen mustard (NM). NEIL1 effectively recognizes and excises the highly mutagenic AFB1-deoxyguanosine adduct in mice, and NEIL1^−/−^ mice have increased sensitivity to AFB1-induced HCC. Both NEIL1 and NEIL3 may protect cells from the mutagenic and cytotoxic effects of NM-formamidopyrimidine; however, NEIL1 may play an important role in initiating BER of AFB1-deoxyguanosine adduct (McCullough and Lloyd, 2019; Vartanian et al., 2017; Minko et al., 2019a).

Additionally, NEIL1 SNP variants are associated with an elevated risk of early onset HCC; in sub-Saharan Africa, patients with the NEIL1 I182M variant are at potential risk of early onset HCC (Zuckerman et al., 2023). The P68H variant showed a slight decrease in efficiency among residents of Qidong County, China, but the A51V and G245R variants showed almost the same activity as the wide type. However, A51V is highly sensitive to temperature, suggesting that its biological activity will be greatly reduced (Minko et al., 2019b).

Similar to NEIL1, NEIL3 is overexpressed in HCC and associated with poor survival. We showed that SNHG3 increases the binding of E2F1 to the promoter region of NEIL3, thereby activating the transcriptional signature of NEIL3 (Zhang et al., 2023). NEIL3 repairs telomere oxidative damage during mitosis and prevents HCC senescence (Zhao et al., 2021b). NEIL3 may promote the migration, invasion, and stemness of HCC cells by activating the BRAF/MEK/ERK/TWIST pathway or by regulating the PI3K/Akt/mTOR signaling pathway (Lai et al., 2022; Wang et al., 2021). A peptide vaccine cocktail derived from NEIL3 has shown initial success in phase I studies of advanced HCC (Ikeda et al., 2021). Several other studies have established prognostic models for HCC involving NEIL3 (Wang et al., 2022a; Hu et al., 2022; Ding et al., 2022; Wu et al., 2022; Wu et al., 2021; Wang et al., 2021a; Lu et al., 2023).

### 4.5 Other cancers

The roles of the NEIL family in various other cancers have been previously reported. CRC is one of the most common cancers worldwide (Dekker et al., 2019; Biller and Schrag, 2021); in CRC, an increase in NEIL1 enhances cell proliferation and reduces apoptosis (Xue et al., 2020); NEIL1 can act as a substrate for the enzymatic deacetylation activity of Sirt3, which may result in the regulation of CRC risk (Kabzinski et al., 2022; Kabziński et al., 2019). However, it remains unclear whether variants in the NEIL family contribute to CRC susceptibility (Dallosso et al., 2008; Broderick et al., 2006). NEIL2 may act as an important marker for predicting radiosensitivity in patients with cancer and is related to overall survival (Kobunai et al., 2011; Pardini et al., 2013; Sayed et al., 2020a; Corral et al., 2013). NEIL3 may also be a prognostic factor for CRC (Jiraskova et al., 2018).

Furthermore, the low activity of NEIL1 caused by mutations and its decreased expression in gastric cancer may be associated with the pathogenesis of few gastric cancers (Goto et al., 2010; Shinmura et al., 2004). *Helicobacter pylori* infection is closely associated with gastric cancer and NEIL2 expression is markedly reduced by *Helicobacter pylori* infection (Sayed et al., 2020b). In addition, the NEIL2 SNP is potentially associated with gastric cancer, esophageal adenocarcinoma, and Barrett’s esophagus risk (Mou et al., 2015; Elingarami et al., 2015; Ali et al., 2022).

The role of the NEIL family in astrocytoma, myeloma, and glioblastoma (Jin et al., 2013; Klattenhoff et al., 2017) and renal clear-cell carcinoma has also been reported (Sousa et al., 2019; Sousa et al., 2013; de Sousa et al., 2017; Sun and Liu, 2023; Peng et al., 2022).

## 5 Concluding remarks/future perspectives

Cellular DNA is constantly exposed to exogenous and endogenous damage, and the accumulation of this damage may result in oxidative DNA damage and strand breaks, thereby affecting genomic integrity and leading to cancer. BER is the primary method used to repair oxidative DNA damage (Iyama and Wilson, 2013). This pathway is initiated by damage-specific DNA glycosylases that recognize and clear damaged bases (Rosenquist et al., 2003; Bj Rås et al., 2017). As a special class of human DNA glycosylases, the NEIL family has a unique structure and a wide range of substrates (Grin and Zharkov, 2011). There is mounting evidence linking members of the NEIL family to cancer occurrence in humans.

As expected given the important role of DNA glycosylases in gene repair, all NEILs directly or indirectly affected cancer characteristics, including cell proliferation, chemoradiotherapy resistance, apoptosis, metastasis, and stemness. This review integrates the mechanisms by which the NEIL family influences the malignant behavior of cancer. Variants in the NEIL family affect cell growth, mainly by affecting the cell cycle. In addition, the abnormal expression of the NEIL family can affect DNA integrity and cause cancer cells to acquire drug resistance and stemness. Notably, NEIL3 can repair telomeres and may play a crucial role in promoting cancer progression.

The expression of members of the NEIL family is regulated by upstream molecules such as RAD9, EP300, and ADAR1. Moreover, post-translational modifications, especially acetylation, significantly influence NEIL expression. ROS levels in cells may also influence the expression of the NEIL family (Fleming et al., 2019).

Possible roles for the NEIL family in many common cancers have been reported. For example, multiple NEIL variants may be associated with cancer susceptibility (Kakhkharova et al., 2022; Zhai et al., 2008; Chae et al., 2016; Wei et al., 2012; Galick et al., 2017; Roy et al., 2007; Prakash et al., 2014). Many studies have listed the NEIL family as potential targets for cancer treatment or as prognostic prediction markers, which may provide suggestions for the precise classification of various cancers and the treatment of drug-resistant cancers (Liao et al., 2022). However, the role of the NEIL family varies in different cancers. Therefore, it is important to identify the pathways involved in the role of the NEIL family in specific cancers. Further comprehensive mechanistic studies are required to confirm these findings.
